# Patterns and drivers of *Nothobranchius* killifish diversity in lowland Tanzania

**DOI:** 10.1002/ece3.8990

**Published:** 2022-06-14

**Authors:** Martin Reichard, Michal Janáč, Radim Blažek, Jakub Žák, Okinyi David Alila, Matej Polačik

**Affiliations:** ^1^ Institute of Vertebrate Biology Academy of Sciences of the Czech Republic Brno Czech Republic; ^2^ Department of Ecology and Vertebrate Zoology University of Łódź Łódź Poland; ^3^ 37748 Department of Botany and Zoology Faculty of Science Masaryk University Brno Czech Republic; ^4^ 37740 Department of Zoology Faculty of Science Charles University Prague Czech Republic; ^5^ 27210 Institute of Ecology and Evolution University of Bern Bern Switzerland; ^6^ Department of Fish Ecology and Evolution Centre of Ecology, Evolution and Biogeochemistry EAWAG Swiss Federal Institute of Aquatic Science and Technology Kastanienbaum Switzerland

**Keywords:** Africa, Cyprinodontiformes, dispersal, ephemeral habitats, habitat protection

## Abstract

Temporary pools are seasonal wetland habitats with specifically adapted biota, including annual *Nothobranchius* killifishes that survive habitat desiccation as diapausing eggs encased in dry sediment. To understand the patterns in the structure of *Nothobranchius* assemblages and their potential in wetland conservation, we compared biodiversity components (alpha, beta, and gamma) between regions and estimated the role and sources of nestedness and turnover on their diversity. We sampled *Nothobranchius* assemblages from 127 pools across seven local regions in lowland Eastern Tanzania over 2 years, using dip net and seine nets. We estimated species composition and richness for each pool, and beta and gamma diversity for each region. We decomposed beta diversity into nestedness and turnover components. We tested nestedness in three main regions (Ruvu, Rufiji, and Mbezi) using the number of decreasing fills metric and compared the roles of pool area, isolation, and altitude on nestedness. A total of 15 species formed assemblages containing 1–6 species. Most *Nothobranchius* species were endemic to one or two adjacent regions. Regional diversity was highest in the Ruvu, Rufiji, and Mbezi regions. Nestedness was significant in Ruvu and Rufiji, with shared core (*N*. *melanospilus*, *N*. *eggersi*, and *N*. *janpapi*) and common (*N. ocellatus* and *N*. *annectens*) species, and distinctive rare species. Nestedness apparently resulted from selective colonization rather than selective extinction, and local species richness was negatively associated with altitude. The *Nothobranchius* assemblages in the Mbezi region were not nested, and had many endemic species and the highest beta diversity driven by species turnover. Overall, we found unexpected local variation in the sources of beta diversity (nestedness and turnover) within the study area. The Mbezi region contained the highest diversity and many endemic species, apparently due to repeated colonizations of the region rather than local diversification. We suggest that annual killifish can serve as a flagship taxon for small wetland conservation.

## Graphical abstract

We compared biodiversity components (alpha, beta, and gamma) of *Nothobranchius* killifish in coastal Tanzanian ecoregions and decomposed beta diversity to nestedness and turnover components. We found unexpected local variation in the sources of beta diversity (nestedness and turnover) within the study area and identified a region that contained the highest diversity and endemism.

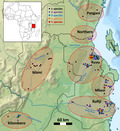

## 1 INTRODUCTION

Freshwater wetlands represent the interface between aquatic and terrestrial habitats, uniquely combining species of both ecosystems. Their extent and broader ecological significance vary seasonally, with annual fluctuation of environmental conditions associated with relatively humid and drier periods. Consequently, only a few species are primarily associated with wetlands and adapted to their dynamic environment (Dalu & Wasserman, 2022; van der Valk, 2012). Wetlands are also a source of immediate and long‐term benefits to human societies, from fishing and livestock support to irrigation and recession agriculture, although many have been seriously degraded (Dalu et al., 2017).

Temporary pools are a common feature of freshwater wetlands (Williams, 2006). They are seasonal habitats and aquatic species may either recolonize each pool during the wet season, or be adapted to persist throughout the wet and dry phases of the annual cycle (Dalu & Wasserman, 2022). Annual recolonization of temporary pools is typical for amphibians, migratory fishes, and flying insects, while local persistence is characteristic of specialized invertebrate taxa, such as large branchiopods, with local egg banks surviving unfavorable conditions as a resting stage (Brock et al., 2003; Vanschoenwinkel et al., 2011). Temporary pools, and especially their resident biota, are ideal candidates for environmental assessment of wetland conditions (Freiry et al., 2020).

*Nothobranchius* is a genus of small freshwater annual killifish of the family Nothobranchiidae (order Cyprinodontiformes; Wildekamp, 2004). The genus is composed of short‐living (3–12 months), sexually dimorphic species (Wildekamp, 2004). Most species reach only 30–70 mm in standard length, with one lineage (containing two species) reaching 10 cm or more (Lambert et al., 2019). *Nothobranchius* fishes are obligatory wetland species, associated with temporary pools (Wildekamp, 2004). Unusually for fish, their life history combines enduring resting stages that survive desiccation, with periods of post‐hatching aquatic life (Reichard & Polačik, 2019). *Nothobranchius* embryos survive the dry part of the annual cycle in a diapausing stage, with minimal metabolism and high capacity to withstand environmental challenges (Polačik et al., 2021). Shortly after habitat inundation, the eggs hatch and *Nothobranchius* fish grow to sexual maturity within a few weeks (Vrtílek et al., 2018). *Nothobranchius* fishes are weak competitors (Reichard, 2015). They are not capable of enduring prolonged competition with fluvial fishes. Their populations are often constrained to isolated (typically rainwater‐fed) pools (Reichard, 2015). Annual killifish dispersal is limited and appears to be a combination of occasional large‐scale flooding and geomorphological changes to the river basins (Bartáková et al., 2015, 2020; van der Merwe et al., 2021).

The conservation status of all 94 currently recognized *Nothobranchius* species has been recently evaluated (Nagy & Watters, 2021). *Nothobranchius* fishes are well characterized taxonomically and contain widespread species and species with locally endemic distribution, ranging from species of least concern to critically endangered (Nagy & Watters, 2021). Several *Nothobranchius* fishes often coexist, forming local assemblages with up to six species in a single temporary pool. Tanzania is the distribution center of the genus, with 47 of the 94 currently known *Nothobranchius* species in Africa, of which many have restricted ranges. The highest species richness is found in coastal Tanzania (van der Merwe et al., 2021). Temporary pools in this region are considerably affected by habitat degradation and loss to human activities, such as urbanization and agriculture expansion (Nagy & Watters, 2021).

To evaluate their possible role as a flagship taxon in temporary wetland conservation and understand the general patterns in the structure of *Nothobranchius* assemblages and their biodiversity, we investigated alpha (species richness in a local pool), beta (compositional differences between local pools within a region), and gamma (regional species richness) diversity and their drivers in 127 temporary pools from seven regions in lowland Tanzania. Specifically, we (1) compared local (alpha) and regional (gamma) diversity of *Nothobranchius* assemblages in particular regions (defined by river catchments) and identified the locally most common species, (2) estimated the level of nestedness in three major regions, (3) tested whether the main source of nestedness was selective extinction or selective colonization, and (4) compared the roles of nestedness and species turnover on beta diversity in the three main regions.

## 2 METHODS

### 2.1 Fish sampling

**FIGURE 1:**
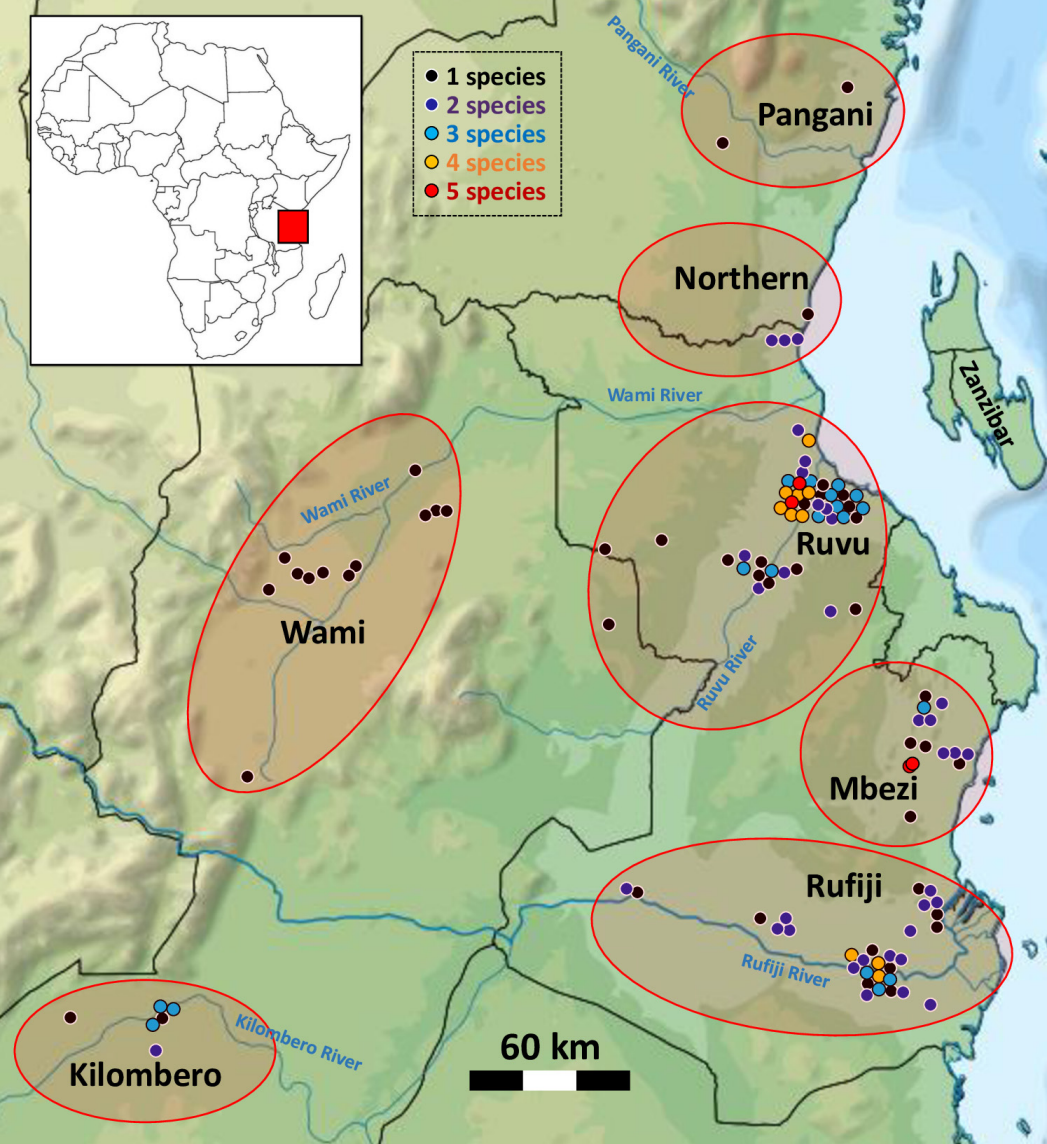
Map of the study area, with sampled pools indicated by dots and grouped into individual regions. Many dots were jittered to enable their visualization, and their GPS coordinates are provided in associated Dryad file (10.5061/dryad.m63xsj44f). Colors denote species richness (black: 1, dark blue: 2, light blue: 3, orange: 4, red: 5 or 6 species)

All data were collected during two field expeditions to lowland Eastern Tanzania. The study area (Figure 1) encompassed the major part of the Coastal ichthyological province (*sensu* Lévêque, 1997), which is formed by the basins of the East African rivers flowing into the Indian Ocean between the Nilo‐Soudanian region in the north and the Zambezi system in the south (Roberts, 1975). The sampling was conducted from May 25 to June 7, 2017, and May 25 to June 11, 2019, at the peak abundance of *Nothobranchius* fishes in the final stage of the main rainy season. The sampling region has two rainy seasons, the main rainy season with more intensive precipitation lasts from March to May and a shorter one from November to mid‐December (Watters et al., 2020). During sampling, we targeted sites with the supposed presence of *Nothobranchius* fishes. In particular, sites were selected on the basis of at least assumed temporal isolation from the main flowing waters. Many of those pools were relatively distant from a stream and were apparently formed by locally drained rainwater rather than inundation from a stream, including pools located in roadside ditches and culverts (which typically represented former natural depressions). Some pools were likely filled with river water during flooding and left isolated after water recession and a few pools still had connections to minor streams. Some sites were active rice paddies.

We collected 185 samples (83 sites in 2017 and 102 sites in 2019). A total of 57 samples (30% of sites) contained no *Nothobranchius* and were not considered in the analyses. One site was found to be located outside the defined study area and was deleted from the final dataset. The final dataset (127 assemblages) contained seven sites with identical geographic position sampled in both years. We considered those samples as independent sampling units, as wetlands form a large inundated area and isolated pools are formed during water recession. Indeed, only in one of these sites were the *Nothobranchius* assemblages identical between the two sampling years. At each site, we recorded GPS location and altitude (Garmin nüvi 550). We estimated pool size and measured its maximum depth to the nearest 5 cm. We used Google Earth software to assign each site to a particular region based on local topography upon reference to a high‐resolution map (Garmin and Google Earth satellite images) and measured the shortest distance to the main regional river. Fish were collected using a dip net with a triangular metal frame (45 × 45 cm) and 5 mm mesh size, with a 1.5 m wooden pole. Typically, 15 to 80 dip‐net hauls were swept at each site but fewer hauls (at least five) were performed in the smallest sites where five hauls already collected most fish individuals from the pool. In larger pools, we used a seine net (length 2.7 m, depth 0.7 m, mesh size 4 mm) in addition to dip‐net sampling. The mesh size used retained adult *Nothobranchius* unselectively and there was no apparent species‐specific bias in the probability of capture (Reichard et al., 2014). Fish were typically unambiguously identified to species in the field and most individuals were returned to the pools. Voucher individuals, or individuals that needed genetic confirmation of species identification (e.g., isolated females which are more difficult to assign to a species), were taken to the laboratory.

Sampling was carried out in accordance with relevant local and international guidelines and regulations. Sample collection complied with legal regulations of Tanzania (research permit: RPGS/R/AS/11/2017) and export of voucher individuals was approved through research associateships with Sokoine University of Agriculture in Morogoro, Tanzania, and associated permit AS/A/1. All data were lawfully acquired in accordance with The Nagoya Protocol on Access to Genetic Resources and the Fair and Equitable Sharing of Benefits Arising from Their Utilization to the Convention on Biological Diversity, with the granted exemption in our case, as the legal procedures to obtain appropriate permissions were not finalized and implemented in the country of data collection (Tanzania) in the years 2017 and 2019, and our research used in situ species determination and release of live individuals.

### 2.2 Data analysis

For each site, we tabulated the presence/absence of each *Nothobranchius* species and associated geographic and environmental variables. For each region, we calculated the mean alpha diversity (mean number of species in a pool, across all sites with at least one *Nothobranchius* species within a particular region), beta diversity (change in assemblage composition across sites, calculated according to Baselga, 2010), and gamma diversity (sum of all species recorded within the region). We then further tested how the assemblages were structured.

To determine nestedness in each of the three main regions (Ruvu, Rufiji, and Mbezi; selected for their sufficient number of sampled assemblages), we used the number of decreasing fills (NODF, Almeida‐Neto et al., 2008) as a nestedness metric. This index can reach values from 0 (completely random) to 100 (perfectly nested). It was suggested as an alternative to metrics such as matrix temperature (Atmar & Patterson, 1993) or discrepancy measure (Brualdi & Sanderson, 1999), with NODF corresponding more properly to a definition of nestedness as a system organization, in which less rich sites/species are subsets of richer sites/species (Almeida‐Neto et al., 2008). Statistical significance was tested by comparing the NODF values with 999 random assemblages created according to a null model. We chose the r1 null model (corresponding to RANDOM1 constraints of Patterson & Atmar, 1986), in which row totals (number of species at a site) remain fixed, with species being “drawn” with probabilities weighted by their observed incidence values (Wright et al., 1997). This model was chosen as a compromise between the equiprobable–equiprobable model, which is truly random, but too prone to type I errors (Ulrich, 2009; Wright et al., 1997) and fixed‐fixed models that preserve both margin totals, which risks being too conservative (Almeida‐Neto et al., 2008). As NODF, similarly to other nestedness metrics, changes with matrix fill (i.e., proportion of 1s in the matrix; Almeida‐Neto et al., 2008) and standardized effect sizes were used to compare nestedness between the three systems studied (Ulrich, 2009).

In the significantly nested systems, we compared the nestedness of sites (i.e. rows), between matrices sorted along gradients of pool area, isolation (measured as a distance from the main river stem), and altitude. Ordering site‐species matrices of the nested systems along gradients of patch area and degree of isolation can help determine whether the system is colonization or extinction driven (Ulrich, 2009). According to Ulrich (2009), extinction‐driven system matrices would be nested along the gradient of area but not isolation, as the colonization in these systems would not be strong enough to generate nestedness. Lack of nestedness in the area‐sorted matrix then would result in rejecting the hypothesis of mass‐effect‐driven nestedness (Ulrich, 2009). We considered alpha level of 0.05 for statistical significance.

Sorting the matrices along the gradient of altitude was further added to our analyses because our experience with the study taxon (Reichard et al., 2017) suggests that species‐specific responses to this parameter can drive nested patterns more than pool area and isolation. As the matrices were ordered along the vertical axis, we searched only for the nestedness of sites, using *N*_rows_ value of NODF (Almeida‐Neto et al., 2008). Each of the ordered matrices within each system was compared to 999 matrices originating by randomly “shuffling” rows of the original matrix. The nestedness analysis was conducted using R 3.5.2 (R Core Team, 2018), using *vegan* library (Oksanen et al., 2013).

## 3 RESULTS

### 3.1 Frequency of species occurrence, shared species, and endemism

**FIGURE 2:**
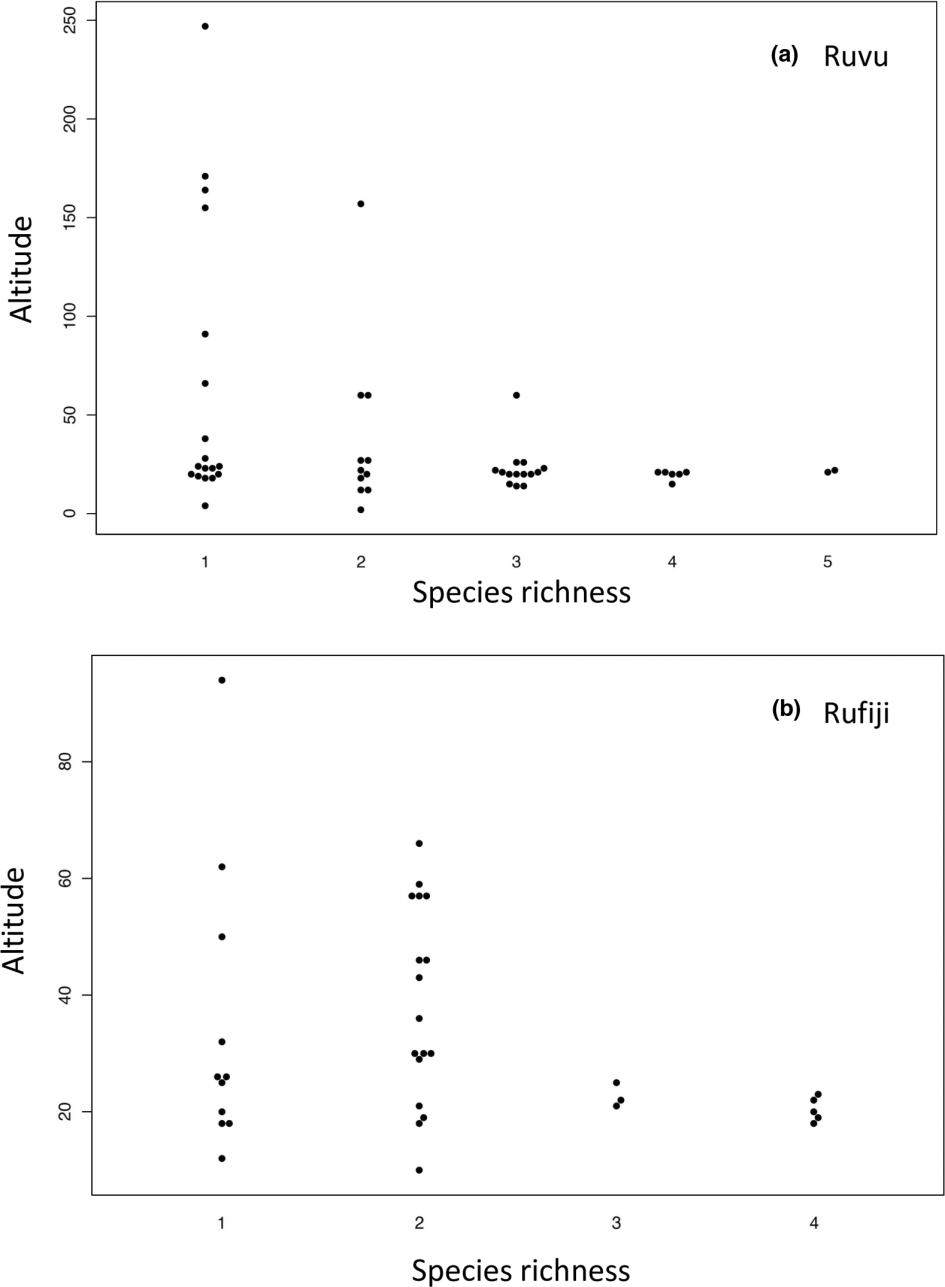
Relationship between *Nothobranchius* species richness and altitude in (a) the Ruvu and (b) Rufiji regions

Overall, 127 of the sampled pools contained at least one *Nothobranchius* species (Figure 1). The Wami region (12 pools with *Nothobranchius* present) contained only a single species (the widespread *N. melanospilus*). *Nothobranchius* pools in the regions Kilombero (seven pools, one to three species), Northern coastal floodplain (four pools, one to two species), and Pangani (two pools, one species in each) were too scarcely represented in our sampling to include in further analyses. *Nothobranchius* assemblages in the other three regions (Ruvu: 47 pools, Rufiji: 36 pools, and Mbezi: 16 pools) were further investigated for their structure, nestedness, beta diversity, and their sources. We detected one to six species coexisting in a pool, with each of the three main regions harboring at least two sites with four *Nothobranchius* species. Local species richness (alpha diversity) was comparable across the three regions and in the Kilombero (Table 1). Higher species richness was found in pools located lower than 30 m above sea level (masl; Figure 2).

**TABLE 1:** List of collected species, with their IUCN conservations status (Nagy & Watters, 2021) and the number of populations detected in particular regions (with the number of *Nothobranchius* assemblages investigated in a region in parentheses)

Species	IUCN	Ruvu (*n* = 51)	Rufiji (*n* = 36)	Mbezi (*n* = 16)	Kilombero (*n* = 7)	Wami (*n* = 12)	North (*n* = 4)	Pangani (*n* = 2)
*N. albimarginatus*	En	–	–	4	–	–	–	–
*N. annectens*	NT	6	6	–	–	–	–	–
*N. eggersi*	LC	28	10	–	–	–	3	–
*N. foerschi*	Vu	2	–	–	–	–	–	–
*N. geminus*	Vu	–	–	–	3	–	–	–
*N. janpapi*	LC	24	17	–	–	–	–	–
*N. lourensi*	NT	–	2	2	6	–	–	–
*N. lucius*	NT	–	–	1	5	–	–	–
*N. luekei*	En	–	–	6	–	–	–	–
*N. melanospilus*	LC	47	33	6	–	12	4	–
*N. ocellatus*	NT	9	6	–	–	–	–	–
*N. palmqvisti*	Vu	–	–	–	–	–	–	1
*N. rubripinnis*	EN	–	–	5	–	–	–	–
*N. ruudwildekampi*	Vu	–	–	7	–	–	–	–
*N. vosseleri*	Vu	–	–	–	–	–	–	1
Alpha diversity	–	2.28	2.00	2.00	2.00	1.00	1.75	1.00
Beta diversity	–	0.77	0.79	0.87	–	–	–	–
Gamma diversity	–	6	6	7	3	2	2	2
Vu + En		2 of 6	0 of 6	6 of 7	3 of 3	0 of 2	0 of 2	2 of 2

Note

For reach region, measures of alpha, beta, and gamma diversity are provided, as well as the sum of species in “Vulnerable” and “Endangered” IUCN categories combined (En: endangered, NT: near threatened, LC: least concern, Vu: vulnerable). Beta diversity was only calculated in three regions with sufficient number of investigated assemblages. Note that we failed to collect two critically endangered species and two vulnerable species reported from the study area.

The Ruvu and Rufiji shared the most species, with *N*. *melanospilus* present in almost all pools containing *Nothobranchius* (92.2% and 91.6% in the Ruvu and Rufiji, respectively). *Nothobranchius eggersi* (54.9% and 27.8%) and *N*. *janpapi* (47.1% and 47.2%) were also common species, followed by predatory *N. ocellatus* (17.6% and 16.7%) and *N*. *annectens* (11.8% and 16.7%). The rarest species (found only in two pools in the regions) differed between the Ruvu (*N*. *foerschi*) and Rufiji (*N*. *lourensi*). The Mbezi region harbored a relatively different and more diverse assemblage of *Nothobranchius* species, with *N*. *ruudwildekampi* (43.8%), *N*. *melanospilus* (37.5%), and *N*. *luekei* (37.5%) the most frequent species. In the Kilombero region, *N*. *lourensi* occurred in most (85.7%) *Nothobranchius* pools. The Mbezi region had five endemic species. Kilombero had one endemic species and both Pangani species were endemic to that region (Table 1). The number of endangered species was highest in the Mbezi region (three of seven species) and all *Nothobranchius* species in the Kilombero and Pangani regions were classified as vulnerable (Table 1).

### 3.2 Nestedness

**FIGURE 3:**
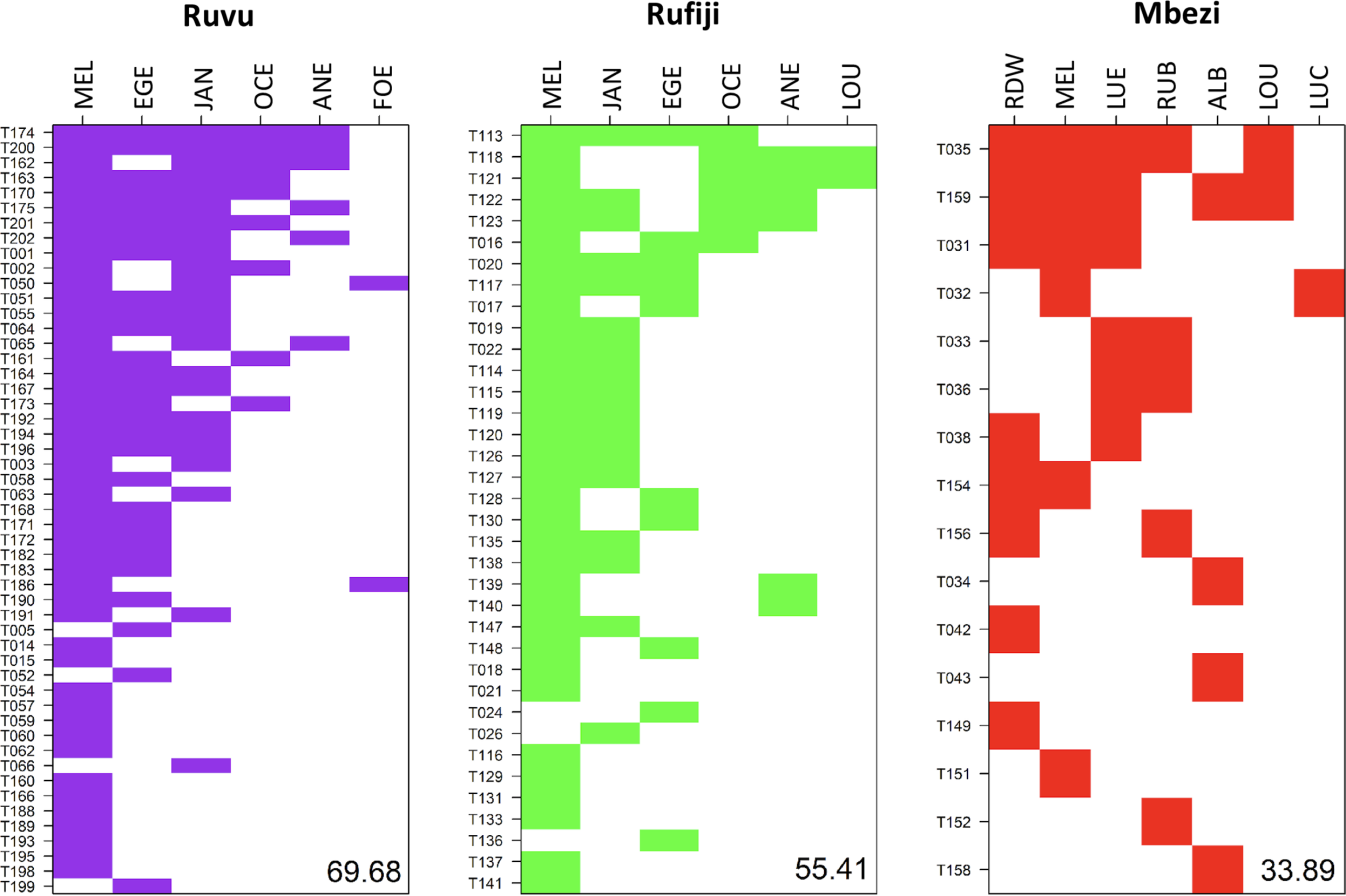
Occurrence of *Nothobranchius* species in three Tanzanian coastal regions visualized as incidence in a packed matrix (i.e., species–sites matrix sorted by species richness and frequency, representing species‐by‐site matrix with minimal nestedness temperature). The NODF values are presented in the bottom right corner of each panel

The Ruvu and Rufiji regions contained significantly nested *Nothobranchius* assemblages (Ruvu: NODF = 69.68; and Rufiji: NODF = 55.41, Figure 3) – their observed NODF differed from randomized matrices (both *p* = .001; Table 2). In contrast, the *Nothobranchius* assemblages in the Mbezi region were not nested (NODF = 33.89, which was not significantly different from randomized matrices; *p* = .766). The standardized effect sizes of NODF were 4.82, 2.97, and −0.75 for the Ruvu, Rufiji, and Mbezi regions, respectively.

**TABLE 2:** Nestedness parameters of *Nothobranchius* assemblages in (a) Ruvu, (b) Rufiji, and (c) Mbezi regions (matrix fill 0.372, 0.362, and 0.187, respectively)

Metric	Value	NM 50%	NM 95%	*p*	SES
(a) Ruvu					
*N*_columns_	64.93	56.44	64.77	.045	1.58
*N*_rows_	69.74	55.94	60.81	.001	4.84
NODF	69.68	55.92	60.73	.001	4.82
(b) Rufiji					
*N*_columns_	54.71	44.27	52.98	.021	1.98
*N*_rows_	55.42	44.74	50.98	.005	2.95
NODF	55.41	44.72	50.91	.005	2.97
(c) Mbezi					
*N*_columns_	39.44	37.14	51.19	.389	0.24
*N*_rows_	32.92	36.81	44.60	.820	−0.91
NODF	33.89	36.68	44.67	.766	−0.75

Note

Metric values calculated for nestedness among species (*N*_columns_), sites (*N*_rows_), and for the whole system are presented, along with their standardized effect sizes (SES), medians, and 95% quantiles of the values calculated for 999 simulated randomized matrices (NM 50%, NM 95%) and the probability (P) that the calculated value exceeds the range of simulated matrices.

### 3.3 The sources of nestedness

**FIGURE 4:**
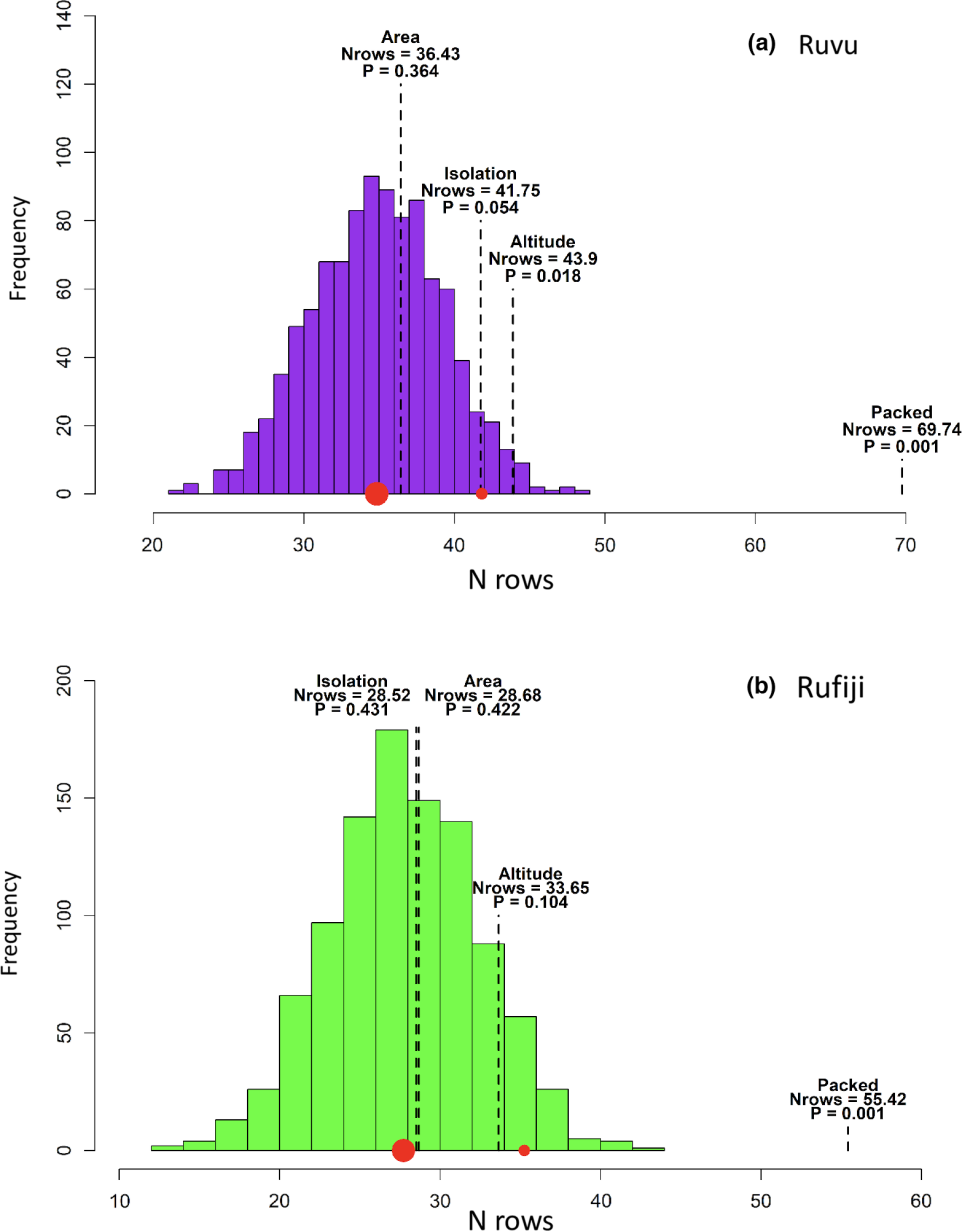
Nestedness of sites (*N*_rows_) for 999 randomly ordered matrices, their median (large red point), and 95% quantile (small red point), and statistics for matrices ordered by area, isolation, and altitude, and for the matrix with minimal nestedness temperature (Packed). *P* = probability that a matrix is more nested than a randomly ordered matrix

Nestedness was not driven by selective extinction because area‐sorted matrices were not associated with nestedness in any region (Ruvu: *N*_rows_ = 36.43, *p* = .364; Rufiji: *N*_rows_ = 28.68, *p* = .422; Figure 4). Selective colonization appeared to have at least a marginal effect on nestedness because the isolation‐sorted matrix had a minor effect on nestedness in the Ruvu region (*N*_rows_ = 41.75, *p* = .054), although not in the Rufiji region (*N*_rows_ = 28.52, *p* = .431). Altitude was found to be the most important factor associated with assemblage nestedness. Its effect was statistically significant in the Ruvu region (*N*_rows_ = 43.90, *p* = .018), but not in the Rufiji region (*N*_rows_ = 33.65, *p* = .104).

### 3.4 The role of nestedness and turnover on beta diversity

**FIGURE 5:**
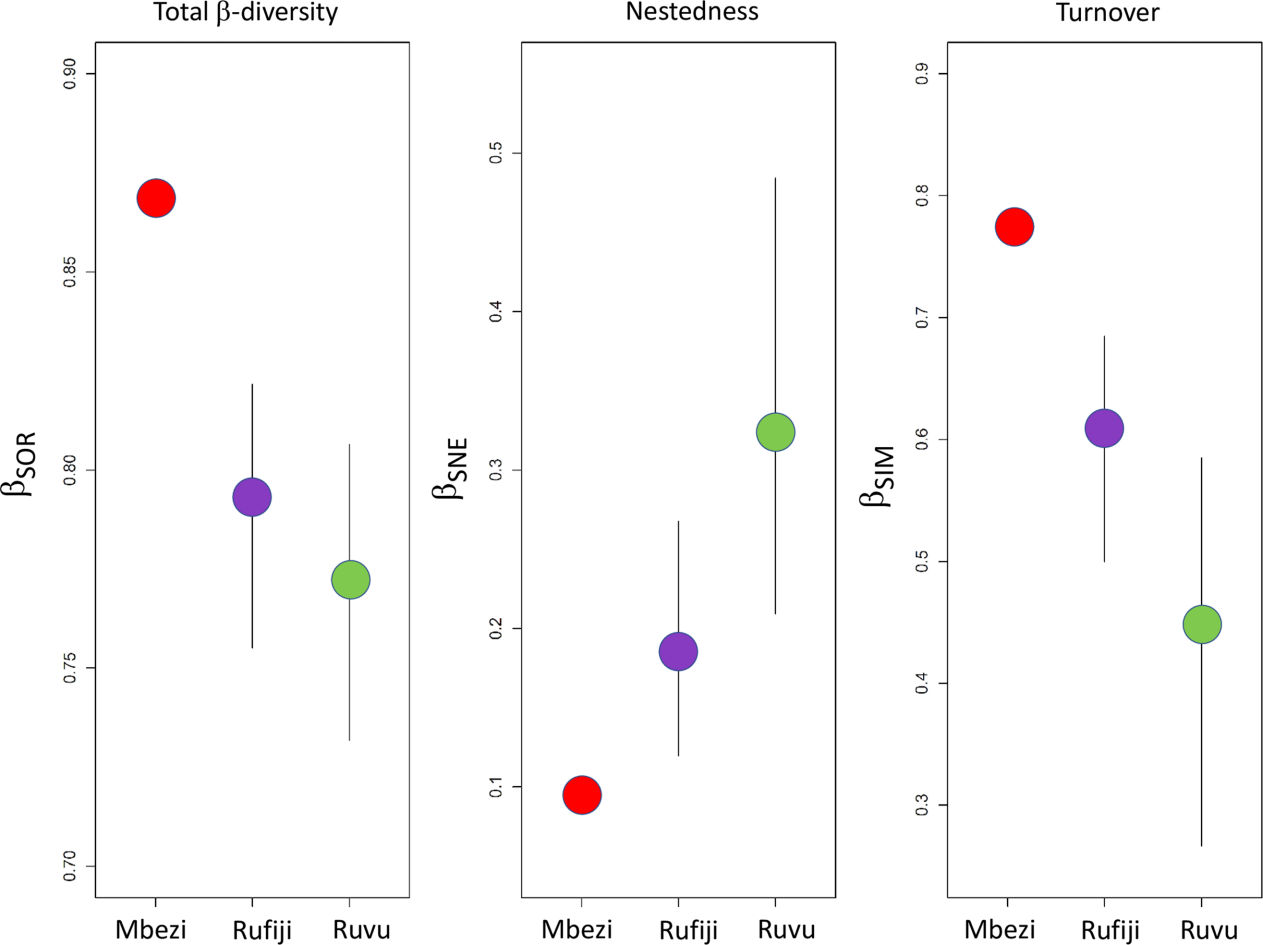
Beta diversity (a) and its components (b: nestedness; c: species turnover) in three study area regions, calculated according to Baselga (2010). Vertical lines represent range of parameters in 1000 random subsets of species‐richer sites, corresponding to the size of the smallest site

The highest beta diversity was found in the Mbezi region, strongly driven by species turnover (Figure 5). The Rufiji and Ruvu regions had similar beta diversity (Figure 5a), but it was primarily driven by nestedness in the Ruvu region and by species turnover in the Rufiji (Figure 5b,c).

## 4 DISCUSSION

*Nothobranchius* are a specialized fish lineage inhabiting temporary pools in African wetlands. We described the patterns of local diversity and assemblage structure in the area where their diversity is globally the highest – lowland Tanzania. We identified region (Mbezi Triangle) where local endemism was the highest and beta diversity was driven by species turnover. In the two spatially largest regions (the basins of the lower Rufiji and lower Ruvu rivers), we found that *Nothobranchius* assemblages were nested, apparently due to selective colonization rather than selective extinction and local species richness was negatively associated with altitude. Most *Nothobranchius* species were endemic to one or two adjacent regions (Table 1), with a single species, *N*. *melanospilus*, occurring across most of the study area.

### 4.1 Regional endemism and species distributions

Our study area formed a major part of the Coastal ichthyological province (*sensu* Lévêque, 1997) in East Africa, which is composed of river basins flowing into the Indian Ocean north of the Zambezi (Roberts, 1975). We identified relatively strong *Nothobranchius* endemism in several regions of this province. In particular, the Mbezi Triangle (composed of minor coastal basins of the Karole, Mbezi, and Luhule, and separated from the Ruvu and Rufiji basins by the Pugu and Mtoti Hills) harbored five endemic species (71% of local species). The Kilombero and Pangani regions (with two of three, and two of two *Nothobranchius* species being endemic to the region, respectively) were also composed of locally isolated species, although with lower species richness. The Ruvu and Rufiji regions shared the major part of their *Nothobranchius* species. These two regions are connected via a swampy area in their middle and upper reaches and are known to share mitochondrial lineages of the widespread *N*. *melanospilus* (Bartáková et al., 2020).

*N*. *melanospilus* was the most common species in the study area, pertaining to all but two regions. This species is locally common and often represents the only *Nothobranchius* species in temporary pools, especially in the Ruvu, Rufiji, and Wami regions. Its exclusive presence was more common in, although not restricted to, higher altitude sites. It is a generalist species (Wildekamp, 2004) with perhaps more effective dispersal ability. *Nothobranchius melanospilus* has been recently divided into three cryptic species (Costa, 2019). While populations assigned to *N*. *kwalensis* occur outside our study area, *N*. *prognathus* was described from the Wami basin. We treat *N*. *melanospilus* as a single species because the validity of the cryptic species has been questioned (Bartáková et al., 2020; Wildekamp, 2019) and is not widely recognized (van der Merwe et al., 2021). If we were to accept the split of *N*. *melanospilus*, the only change in our conclusion would be that *N*. *prognathus* (rather than *N*. *melanospilus*) occurs in the Wami region, and parts of the upper section of the Ruvu region. All other regions apparently contain *N. melanospilus sensu stricto* (Bartáková et al., 2020) and all our main conclusions on assemblage structure would remain unchanged. We acknowledge that a thorough evaluation of *Nothobranchius* diversity on genome‐scale data is needed.

The concept of biological communities posits that some species are naturally very rare (Magurran, 2003), either through their special ecological requirements or particular evolutionary history. We failed to collect four species known from our study area. Two species, *N*. *fuscotaeniatus* and *N*. *steinforti*, are classified as critically endangered, known only from their type localities, and collected only in the years of their discovery (1997 and 1976, respectively; Nagy & Watters, 2021). Our sampling scheme included their type localities and surrounding wetlands, which appear natural and uncompromised, and contained other *Nothobranchius* species. The other two local species not recorded in our samples were *N*. *flammicomantis* (endemic to the Ruvu) and *N*. *kilomberoensis* (endemic to the Kilombero) – both species are not widespread and are classified as vulnerable (Nagy & Watters, 2021), and we may have missed them in our sampling due to interannual variation in their population densities (Nagy & Kiss, 2010; Shidlovsky, 2010).

### 4.2 Local assemblages and sources of their structure

Patterns of beta diversity, and its partitioning into two main components – species turnover and nestedness –, provide an important insight into the structuring of biological communities (Baselga, 2010; Socolar et al., 2016). Local assemblages are shaped by interactions between local and regional processes, which can be neutral (random subsets of regional metacommunity) or deterministic (species sorting according to local conditions; García‐Navas et al., 2022). Hence, species turnover and nestedness reflect different mechanisms underlying the change in assemblage structure across the region – species replacement or their selective loss (Bevilacqua & Terlizzi, 2020).

The highest beta diversity was found in the Mbezi region and arose almost exclusively through species turnover. In contrast, beta diversity in the Ruvu *Nothobranchius* assemblages was driven by nestedness. In the Rufiji region, turnover and nestedness were of intermediate importance. This unexpected local variation in the sources of beta diversity demonstrates that even within the same taxon and spatially constrained study area, the sources of beta diversity can vary substantially. The Mbezi region is most diverse topographically and is composed of small coastal basins interspersed by hills and ridges. While this could make local isolation and evolution of locally endemic species more likely than in the larger floodplains of the Ruvu and Rufiji basins, the phylogenetic hypotheses of the genus (van der Merwe et al., 2021) reject the existence of a locally endemic species flock in the Mbezi region. It appears that repeated colonizations of this region, and perhaps independent speciation events, resulted in the highest local species richness and diversity within the entire *Nothobranchius* range (Lambert et al., 2019; van der Merwe et al., 2021). This can facilitate rapid species turnover at a small geographic scale.

Nestedness describes positive species co‐occurrence resulting in species‐poorer local assemblages being predictable subsets of species‐richer local assemblages (Almeida‐Neto et al., 2008). *Nothobranchius* assemblages were clearly nested in the Ruvu and Rufiji regions. Nested patterns of local assemblages are primarily driven by extinction–colonization dynamics (rather than dispersal and local environmental conditions), and are typically associated with larger spatial scales and higher latitudes (Soininen et al., 2018). For example, significant nestedness in local butterfly assemblages of the Zhoushan archipelago was related to selective extinctions in smaller islands (Xu et al., 2017). Our analysis did not support the hypothesis that *Nothobranchius* assemblage nestedness was driven by selective extinctions. Instead, our data are consistent with the scenario of selective colonization leading to assemblage nestedness. Ephemeral wetland pools are formed within existing floodplain and frequently colonized from adjacent habitat patches at relatively short time scales. In contrast, Zhoushan is a land bridge archipelago, separated from the mainland only 7–9 kya, and local species richness is likely driven by selective extinctions in originally species‐rich assemblages (Xu et al., 2017) over considerably longer time scales than in the wetland ecosystem.

Strong assemblage nestedness reinforces the division into few common and many rare species. Rare species are intrinsically vulnerable and often become endangered (Loiseau et al., 2020). Species rareness may be a function of their evolutionary history (Pepke et al., 2019) or occupation of a specialized niche (Dorado et al., 2011; Markham, 2014). Evolutionary causes of rareness in *Nothobranchius* have been hypothesized in the context of range expansion and contraction effects as a function of evolutionary age (van der Merwe et al., 2021). An example of an ecological source of rareness is the case of a highly derived trophic niche in *N. ocellatus*. This species is a unique predator of other *Nothobranchius* species in the community (Lambert et al., 2019; Watters et al., 2020) and reaches up to 12 cm, two‐ to three‐fold larger than coexisting *Nothobranchius* species. It is typically recorded in assemblages with four *Nothobranchius* species (range 3–5) and apparently requires the presence of congeners that serve as its prey (Wildekamp, 2004). Similar evolution of giant predatory species in annual fish assemblages has been reported from the Neotropics (Helmstetter et al., 2020) and demonstrates that wetland killifish assemblages from various regions converge to a common pattern (García et al., 2009, 2019; Loureiro et al., 2015) through adaptations to specialized ecological niches. While the body morphology of *Nothobranchius* species is relatively conserved (Wildekamp, 2004) and species evolve toward three major body size optima (Lambert et al., 2019), there is preliminary evidence of trophic niche (Polačik et al., 2014; Polačik & Reichard, 2010) and trophic‐related morphology (Costa, 2018; Reichard, 2015) differentiation within local *Nothobranchius* assemblages. A strong phylogenetic hypothesis and comprehensive knowledge of the ecological niche of particular species are needed to understand the role of evolutionary processes in structuring *Nothobranchius* assemblages.

### 4.3 Conservation

The understanding of how nestedness and turnover are linked to beta diversity is important for defining optimal conservation strategies (Socolar et al., 2016). The positive correlation of species turnover with beta diversity is common in tropical assemblages and associated with habitat heterogeneity (Soininen et al., 2018). This pattern is congruent across coexisting functional and taxonomic groups (Gibson et al., 2017), making *Nothobranchius* assemblages a potentially useful proxy for local biodiversity. At the same time, species turnover leads to variation in assemblage composition rather than richness (Hill et al., 2017) across a particular set of assemblages. This highlights the need to conserve larger areas of potentially threatened habitats with a high contribution of species turnover to beta diversity.

Regional pools of *Nothobranchius* species are formed through a combination of ecological and evolutionary processes. The insular nature of seasonal wetland pools supports their isolation (Williams, 2006), including the evolution of many species with small, fragmented geographical ranges (van der Merwe et al., 2021). These range‐restricted species are intrinsically the most endangered (Nagy & Watters, 2021). We have identified the region of highest conservation priority (Mbezi region), where local endemism is highest and beta diversity is driven by species turnover. In the two largest regions (the basins of the lower Rufiji and lower Ruvu rivers), we found that species richness was negatively associated with altitude and that *Nothobranchius* assemblages were nested due to selective colonization rather than selective extinction. Most *Nothobranchius* species were endemic to one or two adjacent regions, with a single species, *N*. *melanospilus*, found across all three major regions.

Although wetlands are now widely recognized as important and valuable ecosystems, they remain largely unprotected and rapidly disappear in Africa and elsewhere (Dalu & Wasserman, 2022; Gardner & Finlayson, 2018). The loss of natural habitat due to agriculture expansion is the greatest threat to wetland fishes in Africa. It contributes to 93% of all species loss followed by natural system modification (38%), resident and commercial development (37%), and pollution (27%; Nagy & Watters, 2021). We have collected multiple species in highly modified habitats, including culverts along the roads and active rice paddies. While this highlights the relative perseverance of *Nothobranchius* populations and communities facing human pressure, it also illustrates the immediate threat to their habitats. Given their bright colors, extensive interest in their collection by hobbyist breeders (Wildekamp, 2004), and their increasing use in biomedical and ecological research (Cellerino et al., 2016), *Nothobranchius* could serve as a flagship group for conservation efforts related to endangered small wetlands. Species‐specific male coloration enables easy and reliable visual identification to species and creates an opportunity for citizen science‐based conservation efforts.

## AUTHOR CONTRIBUTION

**Martin Reichard:** Conceptualization (lead); Data curation (supporting); Formal analysis (supporting); Funding acquisition (lead); Investigation (equal); Methodology (lead); Project administration (lead); Resources (lead); Supervision (lead); Visualization (supporting); Writing – original draft (lead); Writing – review & editing (equal). **Michal Janáč:** Conceptualization (supporting); Data curation (equal); Formal analysis (lead); Investigation (supporting); Methodology (equal); Resources (equal); Visualization (lead); Writing – original draft (supporting); Writing – review & editing (equal). **Radim Blazek:** Conceptualization (supporting); Data curation (supporting); Investigation (equal); Validation (supporting); Visualization (supporting); Writing – review & editing (equal). **Jakub Zak:** Conceptualization (supporting); Investigation (equal); Writing – review & editing (equal). **Okinyi David Alila:** Investigation (equal); Project administration (supporting); Writing – review & editing (equal). **Matej Polačik:** Conceptualization (supporting); Data curation (supporting); Investigation (equal); Methodology (supporting); Writing – review & editing (equal).

## CONFLICT OF INTEREST

The authors declare no competing financial interests.

## Data Availability

Primary data are stored on Dryad (https://doi.org/10.5061/dryad.m63xsj44f).
